# Assessing additive effects of air pollutants on mortality rate in Massachusetts

**DOI:** 10.1186/s12940-021-00704-3

**Published:** 2021-02-23

**Authors:** Yaguang Wei, Brent Coull, Petros Koutrakis, Jiabei Yang, Longxiang Li, Antonella Zanobetti, Joel Schwartz

**Affiliations:** 1 Department of Environmental Health, Harvard T.H. Chan School of Public Health, Landmark Center 4th West, 401 Park Drive, Boston, MA 02215 USA; 2 Department of Biostatistics, Harvard T.H. Chan School of Public Health, Boston, MA USA; 3 Department of Biostatistics, School of Public Health, Brown University, Providence, RI USA; 4 Department of Epidemiology, Harvard T.H. Chan School of Public Health, Boston, MA USA

**Keywords:** Causality, Air pollution, Generalized propensity score, Data aggregation, Scaling random forests

## Abstract

**Background:**

We previously found additive effects of long- and short-term exposures to fine particulate matter (PM_2.5_), ozone (O_3_), and nitrogen dioxide (NO_2_) on all-cause mortality rate using a generalized propensity score (GPS) adjustment approach. The study addressed an important question of how many early deaths were caused by each exposure. However, the study was computationally expensive, did not capture possible interactions and high-order nonlinearities, and omitted potential confounders.

**Methods:**

We proposed two new methods and reconducted the analysis using the same cohort of Medicare beneficiaries in Massachusetts during 2000–2012, which consisted of 1.5 million individuals with 3.8 billion person-days of follow-up. The first method, weighted least squares (WLS), leveraged large volume of data by aggregating person-days, which gave equivalent results to the linear probability model (LPM) method in the previous analysis but significantly reduced computational burden. The second method, m-out-of-n random forests (moonRF), implemented scaling random forests that captured all possible interactions and nonlinearities in the GPS model. To minimize confounding bias, we additionally controlled relative humidity and health care utilizations that were not included previously. Further, we performed low-level analysis by restricting to person-days with exposure levels below increasingly stringent thresholds.

**Results:**

We found consistent results between LPM/WLS and moonRF: all exposures were positively associated with mortality rate, even at low levels. For long-term PM_2.5_ and O_3_, the effect estimates became larger at lower levels. Long-term exposure to PM_2.5_ posed the highest risk: 1 μg/m^3^ increase in long-term PM_2.5_ was associated with 1053 (95% confidence interval [CI]: 984, 1122; based on LPM/WLS methods) or 1058 (95% CI: 988, 1127; based on moonRF method) early deaths each year among the Medicare population in Massachusetts.

**Conclusions:**

This study provides more rigorous causal evidence between PM_2.5_, O_3_, and NO_2_ exposures and mortality, even at low levels. The largest effect estimate for long-term PM_2.5_ suggests that reducing PM_2.5_ could gain the most substantial benefits. The consistency between LPM/WLS and moonRF suggests that there were not many interactions and high-order nonlinearities. In the big data context, the proposed methods will be useful for future scientific work in estimating causality on an additive scale.

**Supplementary Information:**

The online version contains supplementary material available at 10.1186/s12940-021-00704-3.

## Introduction

Ambient fine particulate matter (PM_2.5_), ozone (O_3_), and nitrogen dioxide (NO_2_) are considered leading causes of death worldwide, largely based on associational studies using traditional statistical methods [1–6]. However, such associations do not necessarily indicate causality [7]. Although a growing body of literature has reported the effect of PM_2.5_ exposure on mortality using causal modeling approaches [8–10], few studies so far have examined O_3_ and NO_2_ [11]. Clearly, O_3_ and NO_2_ have received less attention than they deserve; long-term O_3_ concentration has not been regulated by the U.S. National Ambient Air Quality Standards (NAAQS) and the regulations for NO_2_ has been unchanged for decades [12].

Although epidemiologic researchers often report long- or short-term effect of an individual air pollutant, there has been evidence that concurrent air pollution exposures may confound the health effect among each other [2, 3, 13]. In the case of causal analysis, simultaneous assessment of concurrent air pollutants is necessary as it 1) accounts for mutual confounding and thus reduces confounding bias, and 2) allows for comparing the individual effects and identifying the component that is responsible for substantial morbidity and mortality. Indeed, targeting the most harmful air pollutant and its major emission sources based on scientific evidence is the key to efficient and effective air quality regulations [14].

Most air pollution epidemiology studies are conducted using multiplicative models, such as log-linear or Cox proportional hazards models [15]. Such models inherently estimate the effect of exposure on multiplicative scales, which describe the relative change in a health outcome between different exposure levels. In many circumstances, however, it is preferable to measure the absolute effect of exposure on the occurrence of outcome [16]. For example, estimating the additive effect of an air pollutant exposure on mortality rate would give us the number of early deaths due to air pollution, which provides a better sense of the actual size of the health risk and is precisely the type of evidence that U.S. Environmental Protection Agency prefers [17]. In addition, additive models make interaction terms (or their absence) more interpretable, which can help assess effect modification and environmental justice [18].

Recently, we used a parametric generalized propensity score (GPS) adjustment approach to simultaneously estimate causal effects of long- and short-term exposures to PM_2.5_, O_3_, and NO_2_ on mortality rate among Medicare beneficiaries in Massachusetts during 2000–2012 [19]. We considered a counting process for analyzing individual survival data [20]. For each exposure, we estimated the GPS at the observed exposure level on each person-day given the other concurrent exposures and all measured confounders. By modeling the binary outcome of death with linear probability model (LPM), we estimated the additive effect of each exposure on mortality rate. The analysis addressed a critically important question of how many early deaths were caused by air pollution, under the assumption that both GPS model and outcome regression model were correctly specified. However, the GPS models did not capture potential interactions and high-order nonlinearities, making the estimates vulnerable to insufficient confounding control. Further, the counting process data structure with person-day representations of follow-up produces a massive volume of dataset: the whole set of data is comprised of 3.8 billion observations which is about 2 TB in size in the RDS file format, making the analysis computationally expensive.

Using the same cohort, here we proposed two new GPS-based approaches with the goals of increasing computational efficiency and model flexibility in assessing the additive effects of air pollution exposures on mortality. The first approach leveraged the large volume of data by aggregating person-days, which gave the equivalent results to the approach we used in the previous analysis but significantly reduced the computational burden. Building upon the aggregated dataset, the second approach implemented a scaling random forests (RF) method, which increased the flexibility of the GPS model by capturing interactions and nonlinearities. To minimize confounding bias, we also controlled additional community-level confounders that have been suggested as potential confounders. The findings of this analysis will increase the robustness of the association and the validity of causal interpretation of the relationship between air pollution and mortality. In the big data context, the proposed approaches will benefit future scientific work.

## Methods

### Data sources

#### Medicare data

We obtained Medicare enrollment records between January 1, 2000 and December 31, 2012 for beneficiaries aged 65 years and above residing in Massachusetts from the Centers for Medicare and Medicaid Services. We constructed an open cohort with person-day representations of follow-up in which each individual was followed from the maximum of January 1, 2000 or the date of enrollment until death or censoring, whichever occurred earlier. For each beneficiary, we extracted their sex, race/ethnicity, age, Medicaid eligibility, ZIP Code of residence and its latitude and longitude, year of initial enrollment, and date of death if occurred during 2000–2012. Age, Medicaid eligibility, and ZIP Code of residence were updated annually. The outcome of interest is all-cause mortality.

#### Exposure assessment

The daily concentrations of ambient PM_2.5_, O_3_, and NO_2_ at 1 km × 1 km grid cells across the contiguous US were predicted using geographically weighted regressions that ensembled predictions from RF, gradient boosting, and neural network, which integrated multiple data sources including satellite data, land-use variables, monitoring data, chemical transport model simulations, etc. 10-fold cross-validations on held-out monitors indicated good predictive performance, with mean R^2^ of 0.86 for daily PM_2.5_, 0.86 for daily O_3_, and 0.79 for daily NO_2_. Details are published elsewhere [21–23]. The high-resolution and well-validated predictions at 1 km × 1 km grid cells allow us to estimate exposures levels at ZIP Codes with higher degree of accuracy. Using the ZIP Code polygon data generated by Environmental Systems Research Institute [24], for each air pollutant we estimated its daily concentrations in a ZIP Code by averaging the 1 km-gridded predictions with those centroids fall within the boundary of that ZIP Code.

We considered six exposures: long- and short-term exposures to PM_2.5_, O_3_, and NO_2_. For each person on each day, the long-term exposures were defined as annual moving averages of the daily concentrations in the person’s ZIP Code of residence (lag 0–364), and the short-term exposures were defined as two-day moving averages of the daily concentrations in the person’s ZIP Code of residence (lag 0–1). Following the previous literature, the analysis for short-term O_3_ was restricted to person-days in warm season from April to September [3]. The analyses for the other exposures were performed over the entire study period. These exposures were assigned to each person on each day of follow-up.

#### Covariates

We made decisions for confounding selection based on both substantive knowledge and the existing literature [2, 25]. Individual-level covariates, including sex (male or female), race (White, Black, or Other), age group (65–69, 70–74, 75–79, 80–84, or ≥ 85 years), and Medicaid eligibility (as a marker of socioeconomic status), were obtained from the Medicare enrollment records. Daily meteorological covariates, including air surface temperature, dew point temperature, and relative humidity with a resolution of 32 km × 32 km, were obtained from the National Centers for Environmental Prediction/National Center for Atmospheric Research datasets, and were matched to each admission based on the latitude and longitude of the centroid of that person’s ZIP Code of residence [26]. ZIP Code Tabulation Area (ZCTA)-level socioeconomic and housing characteristics of each year, including median household income, median house value, percent of owner-occupied homes, percent of population living in poverty, percent of population below high school education, population density, percent of Blacks, and percent of Hispanics, were linearly interpolated between US Census 2000 and 2010 and were extracted from the American Community Survey for years after 2010, and were matched to each admission based on ZCTA to ZIP Code crosswalks [27]. County-level behavioral factors of each year, including percent of ever smokers, lung cancer rate, and average BMI, were obtained from Behavioral Risk Factor Surveillance System, and were linked to each admission based on the ZIP Code of residence [28]. From the Dartmouth Atlas Project [29], we obtained health care utilization variables including percent of persons over age 65 with an annual hemoglobin A1c test, an annual low-density lipoprotein test, and an annual eye exam in each hospital catchment area, and linked them to each admission based on the ZIP Code of residence. The relative humidity and health care utilization variables were not included in our previous analysis. Because they may confound the association, we added them in the current analysis [30, 31].

### GPS methods for causal Modeling

GPS is a powerful tool for confounding control and is increasingly being used in observational studies [32]. In this section, we presented three GPS-based approaches for assessing the additive effects of long- and short-term exposures to PM_2.5_, O_3_, and NO_2_ on mortality rate. First we reviewed the LPM approach that was used in the previous analysis. Then we presented two new approaches that were developed upon the LPM: weighted least squares (WLS) and m-out-of-n random forests (moonRF). Each approach consisted of two stages: a design stage where GPS were estimated at the observed exposure levels given all measured confounders, and an analysis stage where the additive effects of exposures on mortality rate were estimated conditional upon the estimated GPS [33].

#### Linear probability model (LPM)

We allowed for time-varying covariates by creating a counting process data structure in which each record represents a person-day of follow-up, indexed by *i*. In the design stage, for each exposure we constructed the GPS by fitting a linear regression of the observed exposure level *T*_*i*_ against column vector of covariates ***C***_***i***_:

1 $$ {T}_i={\boldsymbol{C}}_{\boldsymbol{i}}^{\prime}\boldsymbol{\beta} +{\varepsilon}_i, $$

where *ε*_*i*_~*N*(0, *σ*^2^) under the normality assumption, superscript denoted transpose, and ***β*** and *σ* were estimated by ordinal least squares (OLS). The covariate vector ***C***_***i***_ includes the other five exposures, the individual characteristics (sex, race, 5-year age group, and Medicaid eligibility), long- (lag 0–364) and short-term (lag 0–1, lag 2–6, and lag 7–12) moving averages of meteorological variables, the community-level socioeconomic and behavioral variables as mentioned earlier, and calendar year for person-day *i*. Including the other five exposures controlled for jointly confounded exposures; including long- and short-term meteorological variables controlled for confounding of changing weather and climate; and including calendar year controlled for other confounding by time trends. For short-term exposures, we also included calendar month and day of week to control for seasonal confounding. All the continuous covariates were modeled with cubic polynomials to account for potential nonlinearity. A full list of covariates is provided in Section 1 of Additional file 1.

Given the observed exposure *T*_*i*_ and covariates ***C***_***i***_, we estimated the GPS for person-day *i* according to Hirano and Imbens [34]:

2 $$ {\hat{R}}_i=\frac{1}{\sqrt{2\pi {\hat{\sigma}}^2}}\mathit{\exp}\left(-\frac{1}{2{\hat{\sigma}}^2}{\left({T}_i-{\hat{T}}_i\right)}^2\right), $$

where ${\hat{T}}_i={\boldsymbol{C}}_{\boldsymbol{i}}^{\prime}\hat{\boldsymbol{\beta}}$. Assuming that the GPS regression (Eq. 1) had been correctly specified, ${\hat{R}}_i$ was an estimator of *R*_*i*_, which provided a scalar summary of bias introduced by all measured confounders and therefore could be used for confounding control through adjustment.

In the analysis stage, for each exposure we fitted an LPM of binary outcome of death *Y*_*i*_ against *T*_*i*_ and *R*_*i*_:

3 $$ {Y}_i={\alpha}_0+{\alpha}_1{T}_i+{\alpha}_2{R}_i+{\tau}_i, $$

where *α*_0_, *α*_1_, and *α*_2_ were estimated by OLS given ${\hat{R}}_i$ estimated from Eq. 2. Assuming that the GPS model (Eq. 1) and the outcome regression model (Eq. 3) had been correctly specified, the OLS estimate ${\hat{\alpha}}_1$ was an unbiased causal effect estimate, which can be interpreted as the average difference in mortality rate attributed to each unit increase in the exposure. Such causal interpretation comes from the use of GPS and the collapsibility of LPM, making conditional and marginal estimates numerically the same [35, 36]. Due to the heteroscedastic nature of LPM’s residuals [37], we constructed a robust confidence interval (CI) for ${\hat{\alpha}}_1$ using sandwich standard error estimates.

#### Weighted least squares (WLS)

One of the main disadvantages of the LPM method is the challenge in processing the massive dataset with person-day representations of follow-up contributed by the Medicare cohort. Here we proposed the WLS method to reduce the computational burden. The WLS aggregated person-days yet retained all the information after the aggregation. As a result, the WLS gave us the same effect estimates as the LPM but with a significantly improved computational efficiency.

In the design stage, we aggregated the person-days that had the same sex, race, age, Medicaid eligibility, ZIP Code of residence, and date as a single record and assigned the numbers of person-days for that record as weight. This is because the aggregated person-days are identical in terms of all the exposures and covariates, therefore can be treated interchangeably in the analysis. With the aggregated dataset, for each exposure, we fitted a weighted linear regression of the observed exposure level against all the covariates, with continuous ones modeled with cubic polynomials, and estimated the GPS using Eq. 2. We can show that estimating the WLS regression gave the equivalent estimates $\hat{\boldsymbol{\beta}}$ as estimating Eq. 1 using OLS (Section 2 of Additional file 1).

Person-days with the same exposures, covariates, and thus the estimated GPS may have different outcomes of death (0 or 1). In the analysis stage, we calculated the average outcome for each aggregated person-day group and assigned it to the person-day in the aggregated dataset. For each exposure, with the aggregated dataset, we fitted a weighted linear regression of the averaged outcome against the observed exposure level and the estimated GPS. Similarly, estimating this WLS regression gave the equivalent estimates $\hat{\boldsymbol{\alpha}}$ as estimating Eq. 3 (Section 2 of Additional file 1). Hence the WLS produced the same effect estimate ${\hat{\alpha}}_1$ as the LPM.

In our dataset, because most person-days were identical in terms of the exposures and confounders and therefore were dropped after aggregation, the WLS method saved a lot of storage capacity and significantly speeded up the computation; the number of person-days reduced from 3.8 billion to 60 million after data aggregation and compared with the LPM, the computing time reduced from 3 weeks to 2 days.

#### M-out-of-n random forests (moonRF)

RF is a nonparametric learning method for classification or regression which automatically and thoroughly consider possible nonlinear relationship and interactions. They build individual decision trees through intensive resampling and generally yield better predictive performance than linear regression [38]. In the big data context, Bickel et al. [39] proposed a m-out-of-n bootstrap scheme aiming at addressing the computational burden of standard bootstrapping and proved its consistency. The m-out-of-n bootstrap proceeds by resampling *m* observations out of the original dataset (1, …, *n*) without replacement, where *m* ≪ *n*. The number of *m* can be as small as *n*^0.5^, much smaller than the typical size of standard bootstrap samples. Setting the number of bootstrap samples at 50 to 100 obtains fairly good predictive performance, and increasing the number of samples greater than 100 can lead to negligible improvements [38]. Here we estimated the GPS with this idea adopted in the implementation of RF in the design stage of the analysis. The advantage of moonRF over the LPM and WLS is that it has more flexibility to capture any possible interactions and nonlinearities, making the estimates robust to any observed confounding bias [40].

In the design stage, we used the number of person-days aggregated for each record in the aggregated dataset as frequency weight and sampled 62,000 person-days (i.e., *N*^0.5^, where *N* = 3.8 *billion*) without replacement. With this sample, we built a tree for each exposure and made prediction of the exposure for each person-day in the aggregated dataset. We repeated this routine for 100 times. The final predicted exposure level ${\hat{T}}_j$ for person-day *j* was obtained by averaging the predictions of the 100 trees:

4 $$ {\hat{T}}_j=\frac{1}{100}\sum \limits_{l=1}^{100}{\hat{T}}_{jl}. $$

Then applying Eq. 2, we estimated the GPS for each person-day in the aggregated dataset.

In the analysis stage, following the WLS method, we fitted a weighted regression of the averaged outcome against the observed exposure level and the estimated GPS using the aggregated dataset to obtain estimator ${\hat{\alpha}}_1$, which, if both the GPS model and the outcome regression model were correctly specified, was the causal estimate for the additive effect of exposure on mortality rate.

To assess the effects of exposures to low levels of ambient air pollutants, for each method we reconducted the analysis but restricted to person-days with exposure levels below increasingly stringent thresholds, including those well below the levels set in the current NAAQS (12 μg·m^− 3^ for long-term PM_2.5_, 35 μg·m^− 3^ for short-term PM_2.5_, 70 parts per billion [ppb] for short-term O_3_, 53 ppb for long-term NO_2_, and 100 ppb for short-term NO_2_; there is no standard for long-term O_3_).

For each exposure, we estimated annual number of early deaths and the 95% CI attributed to each unit increase in the exposure by multiplying the additive effect estimate ${\hat{\alpha}}_1$ and annual average number of person-days for the study cohort during 2000–2012.

### Sensitivity analyses

We tested the robustness of the main analysis results by conducting sensitivity analyses with respect to the outcome model flexibility (by modeling GPS with cubic polynomial) and the strategy to adjust for seasonality (by including week-of-year and weekday–weekend dummy variables). We also tested the robustness of the moonRF method by increasing bootstrap sample size (up to 620,000) and the number trees (up to 500).

The computations of this study were performed on the Research Computing Environment, supported by the Institute for Quantitative Social Science both in the Faculty of Arts and Sciences at Harvard University. We used R software (version 3.5.1) [41], “ranger” package (version 0.12.1) [42], and “biglm” package (version 0.9.1) [43] to perform the analysis.

## Results

Table 1 shows the descriptive statistics of study population. There were a total of 1,503,572 Medicare beneficiaries in the study. Among those, 561,193 (37.3%) deaths occurred. The population consists of more females (57.5%), mostly whites (92.2%), and mostly aged 65–74 years when entering the cohort (69.0%). 17.0% of the population enrolled in Medicaid. Table 2 summaries the exposure levels across all the beneficiaries’ ZIP Codes of residence during 2000–2012. For each pollutant, the average concentration of long- and short-term exposures were similar while the short-term exposure had greater variation. The exposure levels were mostly below the NAAQS. Descriptive statistics and correlation coefficients among the exposures and covariates are provided in Additional file 1.

**Table 1:** Characteristics of the Medicare population in Massachusetts for the years 2000–2012

	N
Population (%)	1,503,572 (100)
Total person-days	3,874,869,248
Person-days after aggregation	60,708,204
Deaths (%)	561,193 (37.3)
Sex	
Female (%)	864,952 (57.5)
Male (%)	638,620 (42.5)
Race	
White (%)	1,386,883 (92.2)
Black (%)	51,978 (3.5)
Other (%)	64,711 (4.3)
Age at cohort entry	
65–74 (%)	1,037,164 (69.0)
75–84 (%)	335,189 (22.3)
≥ 85 (%)	131,219 (8.7)
Enrollment in Medicaid (%)	255,008 (17.0)

**Table 2:** Summary statistics of air pollution exposures across the ZIP Codes of Medicare beneficiaries’ residence in Massachusetts during 2000–2012

	Long-term PM_2.5_ (μg·m^− 3^) ^a^	Short-term PM_2.5_ (μg·m^− 3^) ^b^	Long-term O_3_ (ppb) ^a^	Short-term O_3_ (ppb) ^b,c^	Long-term NO_2_ (ppb) ^a^	Short-term NO_2_ (ppb) ^b^
Mean ± SD	9.0 ± 1.9	8.9 ± 5.4	37.5 ± 3.0	32.6 ± 10.4	20.4 ± 8.3	20.5 ± 11.6
Min	3.3	0.1	25.7	7.9	3.2	0.2
5th percentile	5.8	3.0	32.2	27.6	8.6	5.3
25th percentile	7.6	5.2	35.6	36.7	14.1	11.3
Median	9.0	7.5	37.7	43.2	19.2	18.7
75th percentile	10.1	11.2	39.6	49.6	26.1	27.9
95th percentile	12.1	19.4	42.1	61.6	35.0	41.6
Max	16.4	65.3	47.1	116.0	64.6	119.0

^a^ Long-term exposure to air pollution was defined as one-year moving average of the exposure level (lag 0–364). ^b^ Short-term exposure to air pollution was defined as two-day moving average of the exposure level (lag 0–1). ^c^ Short-term O_3_ was summarized during the warm season from April 1 to September 30

**Fig. 1:**
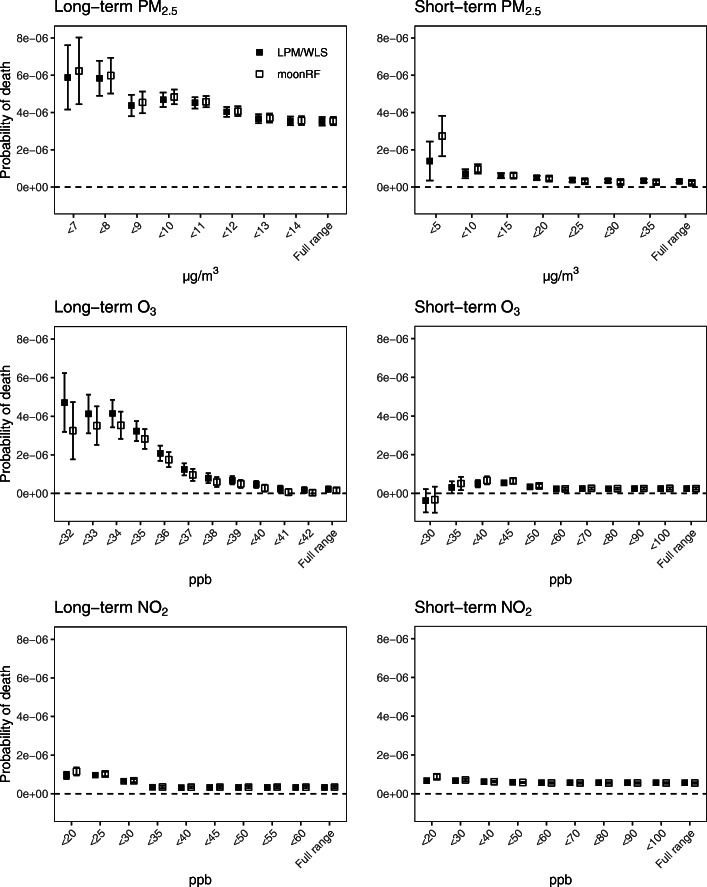
Probabilities of death (and 95% CIs) attribute to 1 μg·m^− 3^ increase in PM_2.5_, 1 ppb increase in O_3_, or 1 ppb increase in NO_2_ at levels below increasingly stringent cutpoints. “Full range” indicates the analysis was performed on the full dataset

Figure 1 shows the results of the three methods at exposure levels below increasingly stringent thresholds. The LPM and WLS methods gave equivalent results and were generally consistent with moonRF. According to the LPM/WLS, with the full dataset, each 1 μg·m^− 3^ increase in long- and short-term exposures to PM_2.5_ was associated with increases of 3.5 × 10^− 6^ (95% CI: 3.3 × 10^− 6^, 3.8 × 10^− 6^) and 3.1 × 10^− 7^ (95% CI: 2.2 × 10^− 7^, 3.9 × 10^− 7^) in the probability of death per person-day, respectively; each 1 ppb increase in long- and short-term exposures to O_3_ was associated with increases of 2.2 × 10^− 7^ (95% CI: 0.8 × 10^− 7^, 3.6 × 10^− 7^) and 2.4 × 10^− 7^ (95% CI: 1.9 × 10^− 7^, 3.0 × 10^− 7^) in the probability of death per person-day, respectively; and each 1 ppb increase in long- and short-term exposures to NO_2_ was associated with increases of 3.3 × 10^− 7^ (95% CI: 2.7 × 10^− 7^, 3.8 × 10^− 7^) and 5.6 × 10^− 7^ (95% CI: 5.2 × 10^− 7^, 6.0 × 10^− 7^) in the probability of death per person-day, respectively. The moonRF estimates were consistent with those of the LPM/WLS on the full dataset. For long-term PM_2.5_ and O_3_, all the methods demonstrated significantly larger effects at lower exposure levels. For short-term O_3_, the LPM/WLS became less consistent with moonRF at very low levels. Numerical values are provided in Section 6 of Additional file 1.

Given the total number of person-days, we estimated the annual number of early deaths due to each exposure (Table 3). With the full dataset, we found that long-term PM_2.5_ was associated with the greatest number of early deaths per unit increase in exposure: the annual number of early deaths associated with 1 μg/m^3^ increase in long-term exposure to PM_2.5_ was 1053 (95% CI: 984, 1122) using LPM/WLS and was 1058 (95% CI: 988, 1127) using moonRF. When restricting the analyses to person-days below the NAAQS, we found greater number of early deaths due to long-term PM_2.5_, short-term PM_2.5_, and short-term O_3_.

**Table 3:** Annual number of early deaths (and 95% CIs) attribute to 1 μg·m^−3^ increase in PM_2.5_, 1 ppb increase in O_3_, or 1 ppb increase in NO_2_

		LPM/WLS	moonRF
Full-range analysis ^a^	Long-term PM_2.5_ (μg·m^−3^)	1053 (984, 1122)	1058 (988, 1127)
	Short-term PM_2.5_ (μg·m^−3^)	92 (67, 117)	69 (44, 95)
	Long-term O_3_ (ppb)	66 (24, 107)	48 (6, 90)
	Short-term O_3_ (ppb)	73 (57, 89)	74 (58, 91)
	Long-term NO_2_ (ppb)	97 (80, 113)	102 (86, 119)
	Short-term NO_2_ (ppb)	167 (156, 179)	163 (151, 174)
Below-standard analysis ^b^	Long-term PM_2.5_ (μg·m^−3^)	1203 (1126, 1280)	1214 (1137, 1292)
	Short-term PM_2.5_ (μg·m^−3^)	101 (74, 127)	78 (52, 105)
	Long-term O_3_ (ppb)	NA	NA
	Short-term O_3_ (ppb)	100 (74, 127)	116 (87, 145)
	Long-term NO_2_ (ppb)	97 (80, 113)	102 (85, 119)
	Short-term NO_2_ (ppb)	168 (156, 179)	163 (151, 174)

^a^ Full-range analysis was performed on the full dataset. ^b^ Below-standard analysis was performed on person-days with exposure levels below the NAAQS (< 12 μg·m^−3^ for long-term PM_2.5_, < 35 μg·m^− 3^ for short-term PM_2.5_, < 70 ppb for short-term O_3_, < 50 ppb for long-term NO_2_, and < 100 ppb for short-term NO_2_; there is no standard for long-term O_3_)

The effect estimates remained robust when fitting the outcome regression with GPS modeled by cubic polynomial, including week-of-year and weekday–weekend dummy variables to adjust for seasonality (Section 7 and 8 of Additional file 1), or increasing the bootstrap sample size and the number of trees in moonRF (Section 9 of Additional file 1).

## Discussion

Building upon the LPM method that we used in the previous analysis [19], we proposed two new GPS-based methods, the WLS and moonRF, to estimate the additive effects of long- and short-term exposures to PM_2.5_, O_3_, and NO_2_ on mortality rate among Medicare beneficiaries in Massachusetts, 2000–2012, encompassing over 3.8 billion person-days of follow-up. Compared with the LPM, the WLS produced identical results but was superior in computational efficiency, whereas the moonRF was superior in flexibility and bias control. To minimize confounding bias, we additionally adjusted for relative humidity and health care utilization variables, which were not included previously. Our results confirmed previous evidence that all the exposures were significantly associated with mortality rate, even at levels below the current NAAQS. For long-term PM_2.5_ and O_3_, the effect sizes were larger when restricting to person-days with exposure levels at increasingly stringent thresholds, suggesting that the exposure-response relationships were nonlinear over full ranges of exposure levels. Using a linear term for each exposure in the outcome regression allowed us to estimate the average difference in mortality rate and further to estimate the number of deaths attributed to a unit increase in the exposure within different ranges of exposure levels. The additive nature of the estimand provides a clearer measure of the health effects of the exposures and is deemed to be of regulatory interest [17]. Comparing the annual number of early deaths associated with each exposure, we found that the long-term PM_2.5_ posed the greatest public health concern, suggesting that reducing PM_2.5_ could potentially gain the most substantial health benefits.

The general consistency between the parametric (LPM/WLS) and nonparametric (moonRF) GPS models is a key finding. Such consistency reduces model dependence while increases the internal validity of the use of GPS for summarizing measured confounding [33]. Some studies, including both conventional statistical and causal modeling analyses, rely on the homogeneity assumption that there are no interaction effects among exposures and confounders [2, 3, 5, 8, 11]. In our study, because the LPM/WLS did not adjust for interactions while the moonRF adjusted for all possible interactions and higher-order nonlinearities, the consistency between the LPM/WLS and moonRF suggests that the homogeneity assumption is likely to hold. In addition, it also suggests that modeling continuous covariates with up to cubic polynomials is sufficient to capture nonlinearities. For long-term O_3_, we found larger difference between the two sets of results at lower levels, which may suggest that the effect was confounded by complex interactions when O_3_ formation was inhibited by lower temperature [22]. It is also possible that NO_2_ was acted as a surrogate as it was inversely related to O_3_ for long-term exposures, and different methods varied in their ability to identify their effects [44]. However, the lack of supporting evidence requires further studies to address this question.

The additive effect estimates provide evidence of the causal relationship between major air pollutants and mortality, which relied on two key assumptions: no unmeasured confounding and positivity [34]. In the observation setting, these two assumptions must always be made to make appropriate causal inference of any public health problems. For the assumption of no unmeasured confounding, although it is impossible to test whether there exists any unobserved confounding, comparing the results with previous literature provide insights into the validity of this assumption. Using a difference-in-difference approach, Wang et al. estimated that a unit increase in annual PM_2.5_ was associated 1.7% increase in mortality rate for people ≥65 years old in New Jersey [10]. Assuming that the baseline mortality was about 5% for the population, their estimate was equivalent to an additive increase of 2.3 × 10^− 6^ in the probability of death per person-day, which was consistent with our estimates (3.5 × 10^− 6^). Such consistency suggests that our long-term effect estimate of PM_2.5_ was not significantly confounded by time-invariant or slowly varying confounders, such as smoking and obesity, since those confounders had been adjusted by design. Similarly, the consistency with a national analysis of short-term PM_2.5_ and NO_2_ with the use of negative exposure control provided additional protection against unmeasured confounding [11]. For the positivity assumption, we cannot prove the lack of positivity with the observed data. Consequently, we categorized each exposure by the lower and upper percentiles and found similar distributions of the estimated GPS across the exposure groups, which suggests that the positivity assumption is likely to hold (Section 10 of Additional file 1). Overall, the consistency with previous studies and the similarity of categorized exposure groups increase the validity of no-unmeasured-confounding and positivity assumptions and, thus, the likelihood of causal connections between the major air pollutants and mortality.

The proposed GPS adjustment approaches have several advantages. First, the use of GPS allows us to adjust for a large number of confounders of both long- and short-term exposures and adequately control for potential nonlinearities and interactions. Because the objective of propensity score estimation is to obtain the best predictive accuracy, we do not need to concern about over-parameterizing. Second, in the analysis stage, the small outcome model with only two covariates, the exposure and the estimated GPS, makes the model fitting and generating robust CIs substantially efficient. Third, the use of OLS regression in the analysis stage also provides a causal interpretation of the exposure coefficient, which comes from the fact that the conditional and marginal estimates are numerically the same.

This study also has limitations. First, although air pollution levels were estimated from models with excellent out-of-sample prediction ability, there is likely measurement error when exposure levels were averaged and assigned to ZIP Codes, which may attenuate effect estimates [45]. While upward bias is also possible, it relies on a combination of large exposure error and high exposure correlation with omitted confounders, which we believe is unlikely [46]. Second, we were not able to adjust for the history of chronic diseases because such information is not available for the Medicare enrollment records, which may leave residual confounding. Third, information bias inherent to the lack of individual-level data, apart from age, sex, race, and Medicaid eligibility, could be present given that ZIP Code was the finest geographical unit we could use to link covariates with each beneficiary.

## Conclusions

Considering the internal validity of the design process for the estimation of GPS and the consistency with previous literature that use several different strategies to address confounding, this study provides more rigorous evidence of the causal relationships between long- and short-term exposures to PM_2.5_, O_3_, and NO_2_ and mortality, even at levels below the current NAAQS. The general consistency between the parametric LPM/WLS and nonparametric moonRF methods suggests that there were not many interaction effects among confounders, and that modeling continuous covariates with up to cubic polynomials was sufficient to capture nonlinearities. In the big data context, the proposed GPS-based methods will be useful in estimating causality on an additive scale for the future scientific work.

## Supplementary Information

**Additional file 1.**

## Data Availability

The exposure data during the current study are available from the corresponding author on reasonable request. The Medicare data are available upon request to the Centers for Medicare and Medicaid Services. The other data are publicly available, with sources described in the manuscript.

## Abbreviations

PM_2.5_: Ambient fine particulate matter
O_3_: Ozone
NO_2_: Nitrogen dioxide
GPS: Generalized propensity score
NAAQS: National Ambient Air Quality Standards
LPM: Linear probability model
WLS: Weighted least squares
moonRF: m-out-of-n random forests
ppb: Parts per billion
