# Supplementary material for: Assessing additive effects of air pollutants on mortality rate in Massachusetts
Source: Environ Health. 2021 Feb 23;20:19. doi: 10.1186/s12940-021-00704-3 (PMC7903765; doi:10.1186/s12940-021-00704-3)
Supplement: Supplementary file 1 — Additional file 1. [file 12940_2021_704_MOESM1_ESM.docx]

**Supplementary Appendix**

*for*

**Assessing additive effects of air pollutants on mortality rate in Massachusetts**

*by*

Yaguang Wei^1^; Brent Coull^2^; Petros Koutrakis^1^; Jiabei Yang^3^; Longxiang Li^1^; Antonella Zanobetti^1^; Joel Schwartz^1,4^

*from*

1. Department of Environmental Health, Harvard T.H. Chan School of Public Health, Boston, MA, USA

2. Department of Biostatistics, Harvard T.H. Chan School of Public Health, Boston, MA, USA

3. Department of Biostatistics, School of Public Health, Brown University, Providence, RI, USA

4. Department of Epidemiology, School of Public Health, Brown University, Providence, RI, USA

**Section 1. List of variables included in main analysis**

**Section 2. Equivalence of LPM and WLS methods**

**Section 3. Descriptive statistics of meteorological and area-level covariates for all ZIP Codes in Massachusetts, 2000–2012**

**Section 4. Pearson correlation coefficients among exposures and meteorological covariates for all ZIP Codes in Massachusetts, 2000–2012**

**Section 5. Pearson correlation coefficients among exposures and area-level covariates for all ZIP Codes in Massachusetts, 2000–2012**

**Section 6. Numeric results of main analysis**

**Section 7. Numeric results of sensitivity analysis of fitting the outcome regression with GPS modeled by cubic polynomial**

**Section 8. Numeric results of sensitivity analysis of including week-of-year and weekday–weekend dummy variables to adjust for seasonality**

**Section 9. Numeric results of sensitivity analysis of increasing bootstrap sample size to 620000 and the number of trees to 500 for moonRF**

**Section 10. Positivity assessment**

# **Section 1. List of variables included in main analysis**

- Short-term PM_2.5_ (lag 0–1)
- Long-term PM_2.5_ (lag 0–364)
- Short-term O_3_ (lag 0–1)
- Long-term O_3_ (lag 0–364)
- Short-term NO_2_ (lag 0–1)
- Long-term NO_2_ (lag 0–364)
- Sex: male or female
- Race: White, Black, or Other
- Age group: 65–69, 70–74, 75–79, 80–84, or ≥ 85 years
- Medicaid eligibility: yes or no
- Air surface temperature over lag 0–1
- Air surface temperature over lag 2–6
- Air surface temperature over lag 7–12
- Air surface temperature over lag 0–364
- Dew point temperature over lag 0–1
- Dew point temperature over lag 2–6
- Dew point temperature over lag 7–12
- Dew point temperature over lag 0–364
- Relative humidity over lag 0–1
- Relative humidity over lag 2–6
- Relative humidity over lag 7–12
- Relative humidity over lag 0–364
- Median household income
- Median house value
- Percent of owner-occupied homes
- Percent below poverty
- Percent of low education
- Population density
- Percent of Blacks
- Percent of Hispanics
- Percent of ever smokers
- Lung cancer rate
- Mean BMI
- Percent of persons over age 65 with an annual HbA1c test
- Percent of persons over age 65 with an annual LDL test
- Percent of persons over age 65 with an annual eye exam
- Year
- Month
- Day of week

# **Section 2. Equivalence of LPM and WLS methods**

Suppose there are $N$ person-days of follow-up in the full dataset (indexed by $i=1,\ldots, N$) and $J$ person-days in the aggregated dataset (indexed by $j=1,\ldots, J$). The number of covariates is $p$.

In the design stage, suppose all the person-days in the full dataset are sorted by exposures and covariates so that duplicate records are brought together in sequence; thus, the exposure vector $\boldsymbol{T}$ and covariate matrix $\boldsymbol{C}$ from the sorted full dataset can be written as:

$\boldsymbol{T}_{\boldsymbol{N\times1}}=\left( \begin{matrix} T_{1} \\ T_{2} \\ \vdots\\ T_{i} \\ \vdots\\ T_{N} \end{matrix} \right)$ and $\boldsymbol{C}_{\boldsymbol{N\times(p+1)}}=\left( \begin{matrix} 1 & C_{11} & C_{12} & \cdots& C_{1p} \\ 1 & C_{21} & C_{22} & \cdots& C_{2p} \\ \vdots& \vdots& \vdots& \cdots& \vdots\\ 1 & C_{i1} & C_{i2} & \cdots& C_{ip} \\ \vdots& \vdots& \vdots& \cdots& \vdots\\ 1 & C_{N1} & C_{N1} & \cdots& C_{Np} \end{matrix} \right)$.

With the joint matrix $(\boldsymbol{T, C})$, after removing all but one of the duplicate observations, the exposure vector $\tilde{\boldsymbol{T}}$ and covariate matrix $\tilde{\boldsymbol{C}}$ can be written as:

${\tilde{\boldsymbol{T}}}_{\boldsymbol{J\times1}}=\left( \begin{matrix} \tilde{T}_{1} \\ \vdots\\ \tilde{T}_{j} \\ \vdots\\ \tilde{T}_{J} \end{matrix} \right)$ and ${\tilde{\boldsymbol{C}}}_{\boldsymbol{J\times(p+1)}}=\left( \begin{matrix} 1 & \tilde{C}_{11} & \cdots& \tilde{C}_{1p} \\ \vdots& \vdots& \cdots& \vdots\\ 1 & \tilde{C}_{j1} & \cdots& \tilde{C}_{jp} \\ \vdots& \vdots& \cdots& \vdots\\ 1 & \tilde{C}_{J1} & \cdots& \tilde{C}_{Jp} \end{matrix} \right)$.

The weight matrix, $\boldsymbol{W}$, is an $J\times J$ diagonal matrix with the number of duplicates for each record $j$ on the diagonal, is written as:

$\boldsymbol{W}_{\boldsymbol{J\times J}}=\left( \begin{matrix} w_{1} & \cdots& 0 \\ \vdots& \ddots& \vdots\\ 0 & \cdots& w_{J} \end{matrix} \right)$.

Using LPM, the OLS estimator $\hat{\boldsymbol{\beta}}$ can be obtained by $\left( \boldsymbol{C}^{\boldsymbol{'}}\boldsymbol{C} \right)^{-1}\boldsymbol{C}^{\boldsymbol{'}}\boldsymbol{T}$, and we can show that

$\boldsymbol{C}^{\boldsymbol{'}}\boldsymbol{C}={\tilde{\boldsymbol{C}}}^{\boldsymbol{'}}\boldsymbol{W}\tilde{\boldsymbol{C}}=\left( \begin{matrix} \sum_{j} w_{j} & \sum_{j} w_{j}\tilde{C}_{j1} & \sum_{j} w_{j}\tilde{C}_{j2} & \cdots& \sum_{j} w_{j}\tilde{C}_{jp} \\ \sum_{j} w_{j}\tilde{C}_{j1} & \sum_{j} w_{j}\tilde{C}_{j1}^{2} & \sum_{j} w_{j}\tilde{C}_{j2}\tilde{C}_{j1} & \cdots& \sum_{j} w_{j}\tilde{C}_{jp}\tilde{C}_{j1} \\ \sum_{j} w_{j}\tilde{C}_{j2} & \sum_{j} w_{j}\tilde{C}_{j1}\tilde{C}_{j2} & \sum_{j} w_{j}\tilde{C}_{j2}^{2} & \cdots& \sum_{j} w_{j}\tilde{C}_{jp}\tilde{C}_{j2} \\ \vdots& \vdots& \vdots& \ddots& \vdots\\ \sum_{j} w_{j}\tilde{C}_{jp} & \sum_{j} w_{j}\tilde{C}_{j1}\tilde{C}_{jp} & \sum_{j} w_{j}\tilde{C}_{j2}\tilde{C}_{jp} & \cdots& \sum_{j} w_{j}\tilde{C}_{jp}^{2} \end{matrix} \right)$ and

$\boldsymbol{C}^{\boldsymbol{'}}\boldsymbol{T}={\tilde{\boldsymbol{C}}}^{\boldsymbol{'}}\boldsymbol{W}\tilde{\boldsymbol{T}}=\left( \begin{matrix} \sum_{j} w_{j}\tilde{T}_{j} \\ \sum_{j} w_{j}\tilde{T}_{j}\tilde{C}_{j1} \\ \sum_{j} w_{j}\tilde{T}_{j}\tilde{C}_{j2} \\ \vdots\\ \sum_{j} w_{j}\tilde{T}_{j}\tilde{C}_{jp} \end{matrix} \right)$.

The two equations above guarantee that LPM and WLS give the equivalent estimator $\hat{\boldsymbol{\beta}}$ in the design stage.

In the analysis stage, suppose the outcome vector $\boldsymbol{Y}$ and design matrix $\boldsymbol{X}$ from the sorted full dataset are:

$\boldsymbol{Y}_{\boldsymbol{N\times1}}=\left( \begin{matrix} Y_{1} \\ Y_{2} \\ \vdots\\ Y_{i} \\ \vdots\\ Y_{N} \end{matrix} \right)$ and $\boldsymbol{X}_{\boldsymbol{N\times3}}=\left( \begin{matrix} 1 & T_{1} & R_{1} \\ 1 & T_{2} & R_{2} \\ \vdots& \vdots& \vdots\\ 1 & T_{i} & R_{i} \\ \vdots& \vdots& \vdots\\ 1 & T_{N} & R_{N} \end{matrix} \right)$,

where $R_{i}$ is the GPS for person-day $i$ at the exposure level $T_{i}$, which has been estimated from the design stage.

Similarly, with the joint matrix $(\boldsymbol{Y, X})$, after removing all but one of the duplicate observations in $\boldsymbol{X}$, the outcome vector $\bar{\boldsymbol{Y}}$ and design matrix $\tilde{\boldsymbol{X}}$ can be written as:

${\bar{\boldsymbol{Y}}}_{\boldsymbol{J\times1}}=\left( \begin{matrix} \bar{Y}_{1} \\ \vdots\\ \bar{Y}_{j} \\ \vdots\\ \bar{Y}_{J} \end{matrix} \right)$ and ${\tilde{\boldsymbol{X}}}_{\boldsymbol{J\times3}}=\left( \begin{matrix} 1 & \tilde{T}_{1} & \tilde{R}_{1} \\ \vdots& \vdots& \vdots\\ 1 & \tilde{T}_{j} & \tilde{R}_{j} \\ \vdots& \vdots& \vdots\\ 1 & \tilde{T}_{J} & \tilde{R}_{J} \end{matrix} \right)$,

where $\bar{Y}_{j}=\frac{1}{w_{j}}\sum_{k=1}^{w_{j}} Y_{jk}$, which is the average outcome among the aggregated person-days for group $j$.

Similarly, using LPM, the OLS estimator $\hat{\boldsymbol{\alpha}}$ can be obtained by $\left( \boldsymbol{X}^{\boldsymbol{'}}\boldsymbol{X} \right)^{-1}\boldsymbol{X}^{\boldsymbol{'}}\boldsymbol{Y}$, and we can show that

$\boldsymbol{X}^{\boldsymbol{'}}\boldsymbol{X}={\tilde{\boldsymbol{X}}}^{\boldsymbol{'}}\boldsymbol{W}\tilde{\boldsymbol{X}}=\left( \begin{matrix} \sum_{j} w_{j} & \sum_{j} w_{j}\tilde{T}_{j} & \sum_{j} w_{j}\tilde{R}_{j} \\ \sum_{j} w_{j}\tilde{T}_{j} & \sum_{j} w_{j}\tilde{T}_{j}^{2} & \sum_{j} w_{j}\tilde{T}_{j}\tilde{R}_{j} \\ \sum_{j} w_{j}\tilde{R}_{j} & \sum_{j} w_{j}\tilde{T}_{j}\tilde{R}_{j} & \sum_{j} w_{j}\tilde{R}_{j}^{2} \end{matrix} \right)$ and

$\boldsymbol{X}^{\boldsymbol{'}}\boldsymbol{Y}=\left( \begin{matrix} \sum_{i} Y_{i} \\ \sum_{i} T_{i}Y_{i} \\ \sum_{i} R_{i}Y_{i} \end{matrix} \right)=\left( \begin{matrix} \sum_{j} w_{j}\bar{Y}_{j} \\ \sum_{j} w_{j}{\tilde{T}_{j}\bar{Y}}_{j} \\ \sum_{j} w_{j}\tilde{R}_{j}\bar{Y}_{j} \end{matrix} \right)={\tilde{\boldsymbol{X}}}^{\boldsymbol{'}}\boldsymbol{W}\bar{\boldsymbol{Y}}$.

The two equations above guarantee that LPM and WLS give the equivalent estimator $\hat{\boldsymbol{\alpha}}$ in the analysis stage.

# **Section 3. Descriptive statistics of meteorological and area-level covariates for all ZIP Codes in Massachusetts, 2000–2012**

| **Covariate** | **Mean** | **SD** |
| --- | --- | --- |
| Air surface temperature over lag 0–1 (Kelvin) | 283.29 | 9.49 |
| Air surface temperature over lag 2–6 (Kelvin) | 283.30 | 9.29 |
| Air surface temperature over lag 7–12 (Kelvin) | 283.31 | 9.26 |
| Air surface temperature over lag 0–364 (Kelvin) | 283.25 | 1.16 |
| Dew point temperature over lag 0–1 (Kelvin) | 279.02 | 9.54 |
| Dew point temperature over lag 2–6 (Kelvin) | 279.03 | 9.17 |
| Dew point temperature over lag 7–12 (Kelvin) | 279.04 | 9.11 |
| Dew point temperature over lag 0–364 (Kelvin) | 279.01 | 1.07 |
| Relative humidity over lag 0–1 (%) | 78.65 | 9.77 |
| Relative humidity over lag 2–6 (%) | 78.64 | 7.71 |
| Relative humidity over lag 7–12 (%) | 78.65 | 7.34 |
| Relative humidity over lag 0–364 (%) | 78.78 | 2.51 |
| Percent of Black (%) | 3.77 | 7.95 |
| Mean BMI (kg/m^2^) | 26.73 | 0.84 |
| Percent of Hispanic (%) | 5.87 | 11.10 |
| Median household income (USD) | 65783.09 | 25590.54 |
| Median house value (USD) | 303688.87 | 158049.41 |
| Percent of persons over age 65 with an annual HbA1c test (%) | 86.55 | 2.48 |
| Percent below poverty (%) | 9.22 | 10.43 |
| Percent of low education (%) | 22.47 | 14.83 |
| Percent of persons over age 65 with an annual eye exam (%) | 76.15 | 2.98 |
| Percent of persons over age 65 with an annual LDL test (%) | 81.23 | 4.03 |
| Percent of owner-occupied homes (%) | 69.57 | 20.09 |
| Lung cancer rate (× 10^4^) | 4.47 | 62.46 |
| Population density (persons/mi^2^) | 3397 | 9032 |
| Percent of ever smokers (%) | 50.28 | 4.45 |

# **Section 4. Pearson correlation coefficients among exposures and meteorological covariates for all ZIP Codes in Massachusetts, 2000–2012**

|  | pm25_ma2 | pm25_ma365 | no2_ma2 | no2_ma365 | ozone_ma2 | ozone_ma365 | air_ma01 | air_ma26 | air_ma712 | air_ma365 | dpt_ma01 | dpt_ma26 | dpt_ma712 | dpt_ma365 | rhum_ma01 | rhum_ma26 | rhum_ma712 | rhum_ma365 |
| --- | --- | --- | --- | --- | --- | --- | --- | --- | --- | --- | --- | --- | --- | --- | --- | --- | --- | --- |
| pm25_ma2 | 1.00 | 0.35 | 0.39 | 0.27 | 0.27 | -0.08 | 0.18 | 0.07 | 0.07 | -0.08 | 0.21 | 0.07 | 0.08 | -0.14 | 0.08 | -0.02 | 0.07 | -0.14 |
| pm25_ma365 |  | 1.00 | 0.54 | 0.75 | -0.06 | -0.23 | -0.04 | -0.04 | -0.04 | -0.21 | -0.04 | -0.05 | -0.05 | -0.34 | -0.07 | -0.08 | -0.09 | -0.34 |
| no2_ma2 |  |  | 1.00 | 0.71 | -0.19 | -0.28 | -0.33 | -0.36 | -0.35 | 0.04 | -0.33 | -0.37 | -0.35 | -0.06 | -0.16 | -0.14 | -0.10 | -0.21 |
| no2_ma365 |  |  |  | 1.00 | -0.11 | -0.40 | 0.00 | 0.00 | 0.00 | 0.05 | -0.01 | -0.01 | -0.01 | -0.07 | -0.06 | -0.08 | -0.08 | -0.28 |
| ozone_ma2 |  |  |  |  | 1.00 | 0.25 | 0.49 | 0.42 | 0.37 | 0.00 | 0.41 | 0.37 | 0.33 | 0.00 | -0.21 | -0.10 | -0.05 | 0.03 |
| ozone_ma365 |  |  |  |  |  | 1.00 | -0.01 | -0.01 | -0.01 | 0.00 | 0.00 | 0.00 | 0.00 | 0.03 | 0.07 | 0.09 | 0.09 | 0.18 |
| air_ma01 |  |  |  |  |  |  | 1.00 | 0.94 | 0.90 | 0.11 | 0.97 | 0.91 | 0.87 | 0.11 | 0.15 | 0.10 | 0.08 | 0.02 |
| air_ma26 |  |  |  |  |  |  |  | 1.00 | 0.94 | 0.12 | 0.89 | 0.98 | 0.92 | 0.12 | 0.06 | 0.14 | 0.09 | 0.02 |
| air_ma712 |  |  |  |  |  |  |  |  | 1.00 | 0.13 | 0.86 | 0.91 | 0.98 | 0.12 | 0.08 | 0.09 | 0.13 | 0.02 |
| air_ma365 |  |  |  |  |  |  |  |  |  | 1.00 | 0.10 | 0.11 | 0.11 | 0.92 | 0.03 | 0.03 | 0.03 | 0.08 |
| dpt_ma01 |  |  |  |  |  |  |  |  |  |  | 1.00 | 0.89 | 0.85 | 0.10 | 0.37 | 0.19 | 0.15 | 0.05 |
| dpt_ma26 |  |  |  |  |  |  |  |  |  |  |  | 1.00 | 0.91 | 0.12 | 0.14 | 0.32 | 0.18 | 0.05 |
| dpt_ma712 |  |  |  |  |  |  |  |  |  |  |  |  | 1.00 | 0.12 | 0.13 | 0.16 | 0.31 | 0.05 |
| dpt_ma365 |  |  |  |  |  |  |  |  |  |  |  |  |  | 1.00 | 0.09 | 0.12 | 0.12 | 0.43 |
| rhum_ma01 |  |  |  |  |  |  |  |  |  |  |  |  |  |  | 1.00 | 0.37 | 0.27 | 0.22 |
| rhum_ma26 |  |  |  |  |  |  |  |  |  |  |  |  |  |  |  | 1.00 | 0.40 | 0.28 |
| rhum_ma712 |  |  |  |  |  |  |  |  |  |  |  |  |  |  |  |  | 1.00 | 0.31 |
| rhum_ma365 |  |  |  |  |  |  |  |  |  |  |  |  |  |  |  |  |  | 1.00 |

# **Section 5. Pearson correlation coefficients among exposures and area-level covariates for all ZIP Codes in Massachusetts, 2000–2012**

|  | pm25_ma2 | pm25_ma365 | no2_ma2 | no2_ma365 | ozone_ma2 | ozone_ma365 | BlkPct | BMI | HspPct | MdHsIcm | MdVlHs | PctA1c | PctAml | PctblPvt | PctblSch | PctEye | PctLDL | Pctmam | PctOwnHs | LungCancerRate | PopDen | smokerate |
| --- | --- | --- | --- | --- | --- | --- | --- | --- | --- | --- | --- | --- | --- | --- | --- | --- | --- | --- | --- | --- | --- | --- |
| pm25_ma2 | 1.00 | 0.35 | 0.39 | 0.27 | 0.27 | -0.08 | 0.07 | -0.09 | 0.07 | -0.03 | -0.04 | -0.17 | -0.17 | 0.07 | 0.10 | -0.16 | -0.24 | -0.18 | -0.13 | 0.00 | 0.09 | -0.10 |
| pm25_ma365 |  | 1.00 | 0.54 | 0.75 | -0.06 | -0.23 | 0.19 | -0.21 | 0.21 | -0.07 | -0.11 | -0.44 | -0.46 | 0.21 | 0.29 | -0.45 | -0.65 | -0.47 | -0.38 | -0.13 | 0.26 | -0.29 |
| no2_ma2 |  |  | 1.00 | 0.71 | -0.19 | -0.28 | 0.22 | -0.13 | 0.20 | 0.00 | 0.07 | -0.19 | -0.33 | 0.17 | 0.16 | -0.30 | -0.31 | -0.30 | -0.35 | -0.01 | 0.25 | -0.28 |
| no2_ma365 |  |  |  | 1.00 | -0.11 | -0.40 | 0.32 | -0.15 | 0.29 | 0.01 | 0.12 | -0.26 | -0.45 | 0.25 | 0.23 | -0.42 | -0.43 | -0.42 | -0.50 | -0.08 | 0.36 | -0.39 |
| ozone_ma2 |  |  |  |  | 1.00 | 0.25 | -0.09 | -0.03 | -0.09 | 0.03 | -0.04 | -0.04 | 0.00 | -0.07 | -0.05 | 0.00 | 0.01 | 0.00 | 0.15 | 0.00 | -0.11 | 0.12 |
| ozone_ma365 |  |  |  |  |  | 1.00 | -0.31 | -0.14 | -0.35 | 0.11 | -0.15 | -0.17 | -0.02 | -0.28 | -0.18 | 0.00 | 0.02 | -0.01 | 0.56 | 0.07 | -0.40 | 0.44 |
| BlkPct |  |  |  |  |  |  | 1.00 | 0.08 | 0.41 | -0.27 | -0.10 | -0.03 | -0.06 | 0.29 | 0.29 | -0.15 | -0.14 | -0.09 | -0.46 | 0.05 | 0.24 | -0.17 |
| BMI |  |  |  |  |  |  |  | 1.00 | 0.17 | 0.04 | 0.09 | 0.12 | 0.13 | 0.07 | 0.09 | -0.04 | 0.24 | 0.01 | -0.04 | -0.01 | -0.05 | -0.12 |
| HspPct |  |  |  |  |  |  |  |  | 1.00 | -0.31 | -0.14 | -0.04 | 0.05 | 0.55 | 0.47 | -0.07 | -0.07 | -0.09 | -0.59 | -0.01 | 0.29 | -0.14 |
| MdHsIcm |  |  |  |  |  |  |  |  |  | 1.00 | 0.66 | 0.12 | -0.19 | -0.17 | -0.43 | -0.01 | 0.12 | 0.00 | 0.50 | 0.05 | -0.10 | -0.23 |
| MdVlHs |  |  |  |  |  |  |  |  |  |  | 1.00 | 0.19 | -0.17 | 0.04 | -0.37 | 0.02 | 0.22 | 0.20 | 0.03 | 0.05 | 0.13 | -0.24 |
| PctA1c |  |  |  |  |  |  |  |  |  |  |  | 1.00 | 0.43 | -0.06 | -0.17 | 0.41 | 0.64 | 0.68 | 0.05 | 0.00 | -0.01 | 0.08 |
| PctAml |  |  |  |  |  |  |  |  |  |  |  |  | 1.00 | -0.02 | -0.12 | 0.52 | 0.48 | 0.51 | 0.10 | 0.00 | -0.12 | 0.34 |
| PctblPvt |  |  |  |  |  |  |  |  |  |  |  |  |  | 1.00 | 0.62 | -0.15 | -0.10 | -0.11 | -0.55 | 0.09 | 0.31 | -0.09 |
| PctblSch |  |  |  |  |  |  |  |  |  |  |  |  |  |  | 1.00 | -0.33 | -0.23 | -0.26 | -0.48 | 0.05 | 0.24 | -0.04 |
| PctEye |  |  |  |  |  |  |  |  |  |  |  |  |  |  |  | 1.00 | 0.41 | 0.56 | 0.20 | 0.00 | -0.18 | 0.29 |
| PctLDL |  |  |  |  |  |  |  |  |  |  |  |  |  |  |  |  | 1.00 | 0.58 | 0.18 | 0.00 | -0.16 | 0.20 |
| Pctmam |  |  |  |  |  |  |  |  |  |  |  |  |  |  |  |  |  | 1.00 | 0.12 | 0.00 | -0.07 | 0.23 |
| PctOwnHs |  |  |  |  |  |  |  |  |  |  |  |  |  |  |  |  |  |  | 1.00 | -0.03 | -0.45 | 0.27 |
| LungCancerRate |  |  |  |  |  |  |  |  |  |  |  |  |  |  |  |  |  |  |  | 1.00 | -0.01 | 0.01 |
| PopDen |  |  |  |  |  |  |  |  |  |  |  |  |  |  |  |  |  |  |  |  | 1.00 | -0.25 |
| smokerate |  |  |  |  |  |  |  |  |  |  |  |  |  |  |  |  |  |  |  |  |  | 1.00 |

# **Section 6. Numeric results of main analysis**

| **WLS** | | | | |
| --- | --- | --- | --- | --- |
|  | **Long-term** | | **Short-term** | |
| PM_2.5_ (µg·m^-3^) | Full range | 3.53e-06 (3.30e-06, 3.76e-06) | Full range | 3.09e-07 (2.24e-07, 3.94e-07) |
|  | <14 | 3.55e-06 (3.31e-06, 3.78e-06) | <35 | 3.38e-07 (2.49e-07, 4.27e-07) |
|  | <13 | 3.67e-06 (3.43e-06, 3.91e-06) | <30 | 3.39e-07 (2.48e-07, 4.30e-07) |
|  | <12 | 4.04e-06 (3.78e-06, 4.30e-06) | <25 | 3.73e-07 (2.77e-07, 4.69e-07) |
|  | <11 | 4.52e-06 (4.22e-06, 4.83e-06) | <20 | 4.98e-07 (3.90e-07, 6.06e-07) |
|  | <10 | 4.69e-06 (4.30e-06, 5.08e-06) | <15 | 6.10e-07 (4.71e-07, 7.48e-07) |
|  | <9 | 4.37e-06 (3.79e-06, 4.95e-06) | <10 | 7.18e-07 (4.77e-07, 9.59e-07) |
|  | <8 | 5.84e-06 (4.90e-06, 6.78e-06) | <5 | 1.39e-06 (3.45e-07, 2.44e-06) |
|  | <7 | 5.88e-06 (4.16e-06, 7.61e-06) |  |  |
| O_3_ (ppb) | Full range | 2.20e-07 (8.07e-08, 3.60e-07) | Full range | 2.44e-07 (1.90e-07, 2.97e-07) |
|  | <42 | 1.72e-07 (1.82e-08, 3.25e-07) | <100 | 2.47e-07 (1.93e-07, 3.01e-07) |
|  | <41 | 2.39e-07 (7.45e-08, 4.04e-07) | <90 | 2.45e-07 (1.91e-07, 3.00e-07) |
|  | <40 | 4.70e-07 (2.86e-07, 6.54e-07) | <80 | 2.38e-07 (1.83e-07, 2.93e-07) |
|  | <39 | 6.77e-07 (4.64e-07, 8.90e-07) | <70 | 2.48e-07 (1.91e-07, 3.07e-07) |
|  | <38 | 7.93e-07 (5.37e-07, 1.05e-06) | <60 | 2.31e-07 (1.65e-07, 2.97e-07) |
|  | <37 | 1.24e-06 (9.31e-07, 1.56e-06) | <50 | 3.37e-07 (2.47e-07, 4.27e-07) |
|  | <36 | 2.08e-06 (1.68e-06, 2.47e-06) | <45 | 5.38e-07 (4.17e-07, 6.60e-07) |
|  | <35 | 3.23e-06 (2.71e-06, 3.74e-06) | <40 | 5.02e-07 (3.16e-07, 6.88e-07) |
|  | <34 | 4.14e-06 (3.43e-06, 4.86e-06) | <35 | 3.09e-07 (-5.56e-09, 6.23e-07) |
|  | <33 | 4.12e-06 (3.11e-06, 5.13e-06) | <30 | -3.79e-07 (-9.82e-07, 2.23e-07) |
|  | <32 | 4.71e-06 (3.19e-06, 6.23e-06) |  |  |
| NO_2_ (ppb) | Full range | 3.25e-07 (2.69e-07, 3.80e-07) | Full range | 5.62e-07 (5.24e-07, 6.00e-07) |
|  | <60 | 3.24e-07 (2.69e-07, 3.80e-07) | <100 | 5.62e-07 (5.24e-07, 6.01e-07) |
|  | <55 | 3.25e-07 (2.69e-07, 3.80e-07) | <90 | 5.62e-07 (5.24e-07, 6.01e-07) |
|  | <50 | 3.24e-07 (2.69e-07, 3.79e-07) | <80 | 5.61e-07 (5.23e-07, 6.00e-07) |
|  | <45 | 3.25e-07 (2.70e-07, 3.81e-07) | <70 | 5.61e-07 (5.22e-07, 5.99e-07) |
|  | <40 | 3.15e-07 (2.59e-07, 3.72e-07) | <60 | 5.66e-07 (5.27e-07, 6.05e-07) |
|  | <35 | 3.31e-07 (2.70e-07, 3.92e-07) | <50 | 5.83e-07 (5.43e-07, 6.23e-07) |
|  | <30 | 6.45e-07 (5.67e-07, 7.23e-07) | <40 | 6.15e-07 (5.70e-07, 6.61e-07) |
|  | <25 | 9.64e-07 (8.50e-07, 1.08e-06) | <30 | 6.64e-07 (5.99e-07, 7.28e-07) |
|  | <20 | 9.51e-07 (7.58e-07, 1.14e-06) | <20 | 6.70e-07 (5.41e-07, 7.99e-07) |
|  | | | | |
| **moonRF** | | | | |
|  | **Long-term** | | **Short-term** | |
| PM_2.5_ (µg·m^-3^) | Full range | 3.55e-06 (3.32e-06, 3.78e-06) | Full range | 2.32e-07 (1.46e-07, 3.18e-07) |
|  | <14 | 3.57e-06 (3.33e-06, 3.80e-06) | <35 | 2.63e-07 (1.73e-07, 3.52e-07) |
|  | <13 | 3.70e-06 (3.46e-06, 3.94e-06) | <30 | 2.67e-07 (1.75e-07, 3.58e-07) |
|  | <12 | 4.07e-06 (3.81e-06, 4.33e-06) | <25 | 3.08e-07 (2.12e-07, 4.04e-07) |
|  | <11 | 4.58e-06 (4.28e-06, 4.89e-06) | <20 | 4.52e-07 (3.45e-07, 5.59e-07) |
|  | <10 | 4.84e-06 (4.45e-06, 5.23e-06) | <15 | 6.21e-07 (4.83e-07, 7.59e-07) |
|  | <9 | 4.55e-06 (3.96e-06, 5.13e-06) | <10 | 9.73e-07 (7.28e-07, 1.22e-06) |
|  | <8 | 5.98e-06 (5.03e-06, 6.94e-06) | <5 | 2.73e-06 (1.65e-06, 3.82e-06) |
|  | <7 | 6.23e-06 (4.44e-06, 8.02e-06) |  |  |
| O_3_ (ppb) | Full range | 1.61e-07 (2.04e-08, 3.02e-07) | Full range | 2.49e-07 (1.95e-07,3.03e-07) |
|  | <42 | 4.16e-08 (-1.12e-07, 1.95e-07) | <100 | 2.50e-07 (1.96e-07, 3.04e-07) |
|  | <41 | 6.74e-08 (-9.60e-08, 2.31e-07) | <90 | 2.52e-07 (1.98e-07, 3.07e-07) |
|  | <40 | 2.80e-07 (9.88e-08, 4.61e-07) | <80 | 2.51e-07 (1.96e-07, 3.06e-07) |
|  | <39 | 4.98e-07 (2.88e-07, 7.08e-07) | <70 | 2.44e-07 (1.88e-07, 2.99e-07) |
|  | <38 | 5.92e-07 (3.40e-07, 8.44e-07) | <60 | 2.53e-07 (1.95e-07, 3.11e-07) |
|  | <37 | 9.60e-07 (6.51e-07, 1.27e-06) | <50 | 2.35e-07 (1.69e-07, 3.02e-07) |
|  | <36 | 1.75e-06 (1.36e-06, 2.14e-06) | <45 | 3.89e-07 (2.90e-07, 4.88e-07) |
|  | <35 | 2.82e-06 (2.31e-06, 3.33e-06) | <40 | 6.42e-07 (5.05e-07, 7.78e-07) |
|  | <34 | 3.53e-06 (2.83e-06, 4.24e-06) | <35 | 6.69e-07 (4.64e-07, 8.74e-07) |
|  | <33 | 3.51e-06 (2.51e-06, 4.51e-06) | <30 | 5.10e-07 (1.61e-07, 8.58e-07) |
|  | <32 | 3.25e-06 (1.77e-06, 4.73e-06) |  |  |
| NO_2_ (ppb) | Full range | 3.43e-07 (2.87e-07, 3.98e-07) | Full range | 5.46e-07 (5.08e-07, 5.85e-07) |
|  | <60 | 3.42e-07 (2.87e-07, 3.98e-07) | <100 | 5.47e-07 (5.08e-07, 5.85e-07) |
|  | <55 | 3.43e-07 (2.87e-07, 3.98e-07) | <90 | 5.47e-07 (5.08e-07, 5.86e-07) |
|  | <50 | 3.42e-07 (2.86e-07, 3.98e-07) | <80 | 5.46e-07 (5.07e-07, 5.85e-07) |
|  | <45 | 3.43e-07 (2.88e-07, 3.99e-07) | <70 | 5.45e-07 (5.07e-07, 5.84e-07) |
|  | <40 | 3.36e-07 (2.78e-07, 3.93e-07) | <60 | 5.51e-07 (5.12e-07, 5.90e-07) |
|  | <35 | 3.53e-07 (2.91e-07, 4.15e-07) | <50 | 5.72e-07 (5.32e-07, 6.12e-07) |
|  | <30 | 6.72e-07 (5.92e-07, 7.52e-07) | <40 | 6.20e-07 (5.75e-07, 6.66e-07) |
|  | <25 | 1.02e-06 (9.01e-07, 1.14e-06) | <30 | 7.03e-07 (6.39e-07, 7.68e-07) |
|  | <20 | 1.15e-06 (9.51e-07, 1.36e-06) | <20 | 8.71e-07 (7.40e-07, 1.00e-06) |

Numbers represent the probabilities of death (and 95% CIs) attribute to 1 µg·m^-3^ increase in PM_2.5_, 1 ppb increase in O_3_, or 1 ppb increase in NO_2_ at levels below increasingly stringent thresholds.

# **Section 7. Numeric results of sensitivity analysis of fitting the outcome regression with GPS modeled by cubic polynomial**

| **WLS** | | | | |
| --- | --- | --- | --- | --- |
|  | **Long-term** | | **Short-term** | |
| PM_2.5_ (µg·m^-3^) | Full range | 3.53e-06 (3.30e-06, 3.76e-06) | Full range | 3.09e-07 (2.24e-07, 3.94e-07) |
|  | <14 | 3.55e-06 (3.31e-06, 3.78e-06) | <35 | 3.38e-07 (2.49e-07, 4.27e-07) |
|  | <13 | 3.67e-06 (3.43e-06, 3.91e-06) | <30 | 3.37e-07 (2.48e-07, 4.30e-07) |
|  | <12 | 4.04e-06 (3.78e-06, 4.30e-06) | <25 | 3.73e-07 (2.77e-07, 4.69e-07) |
|  | <11 | 4.52e-06 (4.22e-06, 4.83e-06) | <20 | 4.98e-07 (3.90e-07, 6.06e-07) |
|  | <10 | 4.69e-06 (4.30e-06, 5.08e-06) | <15 | 6.10e-07 (4.71e-07, 7.48e-07) |
|  | <9 | 4.37e-06 (3.79e-06, 4.95e-06) | <10 | 7.18e-07 (4.77e-07, 9.59e-07) |
|  | <8 | 5.84e-06 (4.90e-06, 6.78e-06) | <5 | 1.39e-06 (3.45e-07, 2.44e-06) |
|  | <7 | 5.88e-06 (4.16e-06, 7.61e-06) |  |  |
| O_3_ (ppb) | Full range | 2.20e-07 (8.07e-08, 3.60e-07) | Full range | 2.44e-07 (1.90e-07, 2.97e-07) |
|  | <42 | 1.72e-07 (1.82e-08, 3.25e-07) | <100 | 2.47e-07 (1.93e-07, 3.01e-07) |
|  | <41 | 2.39e-07 (7.45e-08, 4.04e-07) | <90 | 2.45e-07 (1.91e-07, 2.30e-07) |
|  | <40 | 4.70e-07 (2.86e-07, 6.54e-07) | <80 | 2.38e-07 (1.83e-07, 2.93e-07) |
|  | <39 | 6.77e-07 (4.64e-07, 8.90e-07) | <70 | 2.49e-07 (1.91e-07, 3.07e-07) |
|  | <38 | 7.93e-07 (5.37e-07, 1.05e-06) | <60 | 2.31e-07 (1.65e-07, 2.97e-07) |
|  | <37 | 1.24e-06 (9.31e-07, 1.56e-06) | <50 | 3.37e-07 (2.47e-07, 4.27e-07) |
|  | <36 | 2.08e-06 (1.68e-06, 2.47e-06) | <45 | 5.38e-07 (4.17e-07, 6.60e-07) |
|  | <35 | 3.23e-06 (2.71e-06, 3.74e-06) | <40 | 5.02e-07 (3.16e-07, 6.88e-07) |
|  | <34 | 4.14e-06 (3.43e-06, 4.86e-06) | <35 | 3.09e-07 (-5.56e-09, 6.23e-07) |
|  | <33 | 4.12e-06 (3.11e-06, 5.13e-06) | <30 | -3.79e-07 (-9.82e-07, 2.23e-07) |
|  | <32 | 4.71e-06 (3.19e-06, 6.23e-06) |  |  |
| NO_2_ (ppb) | Full range | 3.25e-07 (2.69e-07, 3.80e-07) | Full range | 5.62e-07 (5.24e-07, 6.00e-07) |
|  | <60 | 3.24e-07 (2.69e-07, 3.80e-07) | <100 | 5.62e-07 (5.24e-07, 6.01e-07) |
|  | <55 | 3.25e-07 (2.69e-07, 3.80e-07) | <90 | 5.62e-07 (5.24e-07, 6.01e-07) |
|  | <50 | 3.24e-07 (2.69e-07, 3.79e-07) | <80 | 5.61e-07 (5.23e-07, 6.00e-07) |
|  | <45 | 3.25e-07 (2.70e-07, 3.81e-07) | <70 | 5.61e-07 (5.22e-07, 5.99e-07) |
|  | <40 | 3.15e-07 (2.59e-07, 3.72e-07) | <60 | 5.66e-07 (5.27e-07, 6.05e-07) |
|  | <35 | 3.31e-07 (2.70e-07, 3.92e-07) | <50 | 5.83e-07 (5.43e-07, 6.23e-07) |
|  | <30 | 6.45e-07 (5.67e-07, 7.23e-07) | <40 | 6.15e-07 (5.70e-07, 6.61e-07) |
|  | <25 | 9.64e-07 (8.50e-07, 1.08e-06) | <30 | 6.64e-07 (5.99e-07, 7.28e-07) |
|  | <20 | 9.51e-07 (7.58e-07, 1.14e-06) | <20 | 6.70e-07 (5.41e-07, 7.99e-07) |
|  | | | | |
| **moonRF** | | | | |
|  | **Long-term** | | **Short-term** | |
| PM_2.5_ (µg·m^-3^) | Full range | 3.55e-06 (3.32e-06, 3.78e-06) | Full range | 2.32e-07 (1.46e-07, 3.18e-07) |
|  | <14 | 3.57e-06 (3.33e-06, 3.80e-06) | <35 | 2.63e-07 (1.73e-07, 3.52e-07) |
|  | <13 | 3.70e-06 (3.46e-06, 3.94e-06) | <30 | 2.67e-07 (1.75e-07, 3.58e-07) |
|  | <12 | 4.07e-06 (3.81e-06, 4.33e-06) | <25 | 3.08e-07 (2.12e-07, 4.04e-07) |
|  | <11 | 4.58e-06 (4.28e-06, 4.89e-06) | <20 | 4.52e-07 (3.45e-07, 5.59e-07) |
|  | <10 | 4.84e-06 (4.45e-06, 5.23e-06) | <15 | 6.21e-07 (4.83e-07, 7.59e-07) |
|  | <9 | 4.55e-06 (3.96e-06, 5.13e-06) | <10 | 9.73e-07 (7.28e-07, 1.22e-06) |
|  | <8 | 5.98e-06 (5.03e-06, 6.94e-06) | <5 | 2.73e-06 (1.65e-06, 3.82e-06) |
|  | <7 | 6.23e-06 (4.44e-06, 8.02e-06) |  |  |
| O_3_ (ppb) | Full range | 1.61e-07 (2.04e-08, 3.02e-07) | Full range | 2.50e-07 (1.96e-07, 3.04e-07) |
|  | <42 | 4.16e-08 (-1.12e-07, 1.95e-07) | <100 | 2.52e-07 (1.98e-07, 3.07e-07) |
|  | <41 | 6.74e-08 (-9.60e-08, 2.31e-07) | <90 | 2.51e-07 (1.96e-07, 3.06e-07) |
|  | <40 | 2.80e-07 (9.88e-08, 4.61e-07) | <80 | 2.44e-07 (1.88e-07, 2.99e-07) |
|  | <39 | 4.98e-07 (2.88e-07, 7.08e-07) | <70 | 2.53e-07 (1.95e-07, 3.11e-07) |
|  | <38 | 5.92e-07 (3.40e-07, 8.44e-07) | <60 | 2.35e-07 (1.69e-07, 3.02e-07) |
|  | <37 | 9.60e-07 (6.51e-07, 1.27e-06) | <50 | 3.89e-07 (2.90e-07, 4.88e-07) |
|  | <36 | 1.75e-06 (1.36e-06, 2.14e-06) | <45 | 6.42e-07 (5.05e-07, 7.78e-07) |
|  | <35 | 2.82e-06 (2.31e-06, 3.33e-06) | <40 | 6.69e-07 (4.64e-07, 8.74e-07) |
|  | <34 | 3.53e-06 (2.83e-06, 4.24e-06) | <35 | 5.10e-07 (1.61e-07, 8.58e-07) |
|  | <33 | 3.51e-06 (2.51e-06, 4.51e-06) | <30 | -3.24e-07 (-9.90e-07, 3.43e-07) |
|  | <32 | 3.25e-06 (1.77e-06, 4.73e-06) |  |  |
| NO_2_ (ppb) | Full range | 3.43e-07 (2.87e-07, 3.98e-07) | Full range | 5.46e-07 (5.08e-07, 5.85e-07) |
|  | <60 | 3.42e-07 (2.87e-07, 3.98e-07) | <100 | 5.47e-07 (5.08e-07, 5.85e-07) |
|  | <55 | 3.43e-07 (2.87e-07, 3.98e-07) | <90 | 5.47e-07 (5.08e-07, 5.86e-07) |
|  | <50 | 3.42e-07 (2.86e-07, 3.98e-07) | <80 | 5.46e-07 (5.07e-07, 5.85e-07) |
|  | <45 | 3.43e-07 (2.88e-07, 3.99e-07) | <70 | 5.45e-07 (5.07e-07, 5.84e-07) |
|  | <40 | 3.36e-07 (2.78e-07, 3.93e-07) | <60 | 5.51e-07 (5.12e-07, 5.90e-07) |
|  | <35 | 3.53e-07 (2.91e-07, 4.15e-07) | <50 | 5.72e-07 (5.32e-07, 6.12e-07) |
|  | <30 | 6.72e-07 (5.92e-07, 7.52e-07) | <40 | 6.20e-07 (5.75e-07, 6.66e-07) |
|  | <25 | 1.02e-06 (9.01e-07, 1.14e-06) | <30 | 7.03e-07 (6.39e-07, 7.68e-07) |
|  | <20 | 1.15e-06 (9.51e-07, 1.36e-06) | <20 | 8.71e-07 (7.40e-07, 1.00e-06) |

Numbers represent the probabilities of death (and 95% CIs) attribute to 1 µg·m^-3^ increase in PM_2.5_, 1 ppb increase in O_3_, or 1 ppb increase in NO_2_ at levels below increasingly stringent thresholds.

# **Section 8. Numeric results of sensitivity analysis of including week-of-year and weekday–weekend dummy variables to adjust for seasonality**

| **WLS** | | | | |
| --- | --- | --- | --- | --- |
|  | **Long-term** | | **Short-term** | |
| PM_2.5_ (µg·m^-3^) | Full range | 3.53e-06 (3.30e-06, 3.76e-06) | Full range | 3.10e-07 (2.25e-07, 3.95e-07) |
|  | <14 | 3.55e-06 (3.31e-06, 3.78e-06) | <35 | 3.40e-07 (2.51e-07, 4.29e-07) |
|  | <13 | 3.67e-06 (3.43e-06, 3.91e-06) | <30 | 3.40e-07 (2.49e-07, 4.32e-07) |
|  | <12 | 4.04e-06 (3.78e-06, 4.30e-06) | <25 | 3.74e-07 (2.78e-07, 4.70e-07) |
|  | <11 | 4.52e-06 (4.22e-06, 4.82e-06) | <20 | 5.00e-07 (3.92e-07, 6.08e-07) |
|  | <10 | 4.69e-06 (4.30e-06, 5.08e-06) | <15 | 6.12e-07 (4.73e-07, 7.50e-07) |
|  | <9 | 4.37e-06 (3.79e-06, 4.95e-06) | <10 | 7.18e-07 (4.77e-07, 9.60e-07) |
|  | <8 | 5.84e-06 (4.90e-06, 6.78e-06) | <5 | 1.36e-06 (3.11e-07, 2.41e-06) |
|  | <7 | 5.89e-06 (4.16e-06, 7.61e-06) |  |  |
| O_3_ (ppb) | Full range | 2.21e-07 (8.13e-08, 3.61e-07) | Full range | 2.43e-07 (1.89e-07, 2.96e-07) |
|  | <42 | 1.74e-07 (2.02e-08, 3.27e-07) | <100 | 2.46e-07 (1.92e-07, 3.00e-07) |
|  | <41 | 2.42e-07 (7.70e-08, 4.06e-07) | <90 | 2.44e-07 (1.90e-07, 2.99e-07) |
|  | <40 | 4.73e-07 (2.89e-07, 6.57e-07) | <80 | 2.37e-07 (1.82e-07, 2.93e-07) |
|  | <39 | 6.80e-07 (4.67e-07, 8.93e-07) | <70 | 2.48e-07 (1.90e-07, 3.06e-07) |
|  | <38 | 7.96e-07 (5.41e-07, 1.05e-06) | <60 | 2.32e-07 (1.66e-07, 2.97e-07) |
|  | <37 | 1.25e-06 (9.34e-07, 1.56e-06) | <50 | 3.40e-07 (2.50e-07, 4.30e-07) |
|  | <36 | 2.08e-06 (1.69e-06, 2.48e-06) | <45 | 5.42e-07 (4.20e-07, 6.64e-07) |
|  | <35 | 3.23e-06 (2.71e-06, 3.74e-06) | <40 | 5.07e-07 (3.21e-07, 6.93e-07) |
|  | <34 | 4.14e-06 (3.43e-06, 4.86e-06) | <35 | 3.18e-07 (3.74e-09, 6.33e-07) |
|  | <33 | 4.13e-06 (3.12e-06, 5.14e-06) | <30 | -3.79e-07 (-9.82e-07, 2.24e-07) |
|  | <32 | 4.74e-06 (3.22e-06, 6.26e-06) |  |  |
| NO_2_ (ppb) | Full range | 3.25e-07 (2.69e-07, 3.80e-07) | Full range | 5.61e-07 (5.23e-07, 6.00e-07) |
|  | <60 | 3.24e-07 (2.69e-07, 3.80e-07) | <100 | 5.62e-07 (5.23e-07, 6.00e-07) |
|  | <55 | 3.25e-07 (2.69e-07, 3.80e-07) | <90 | 5.62e-07 (5.23e-07, 6.00e-07) |
|  | <50 | 3.24e-07 (2.69e-07, 3.79e-07) | <80 | 5.61e-07 (5.22e-07, 5.99e-07) |
|  | <45 | 3.25e-07 (2.70e-07, 3.81e-07) | <70 | 5.60e-07 (5.22e-07, 5.99e-07) |
|  | <40 | 3.15e-07 (2.59e-07, 3.72e-07) | <60 | 5.65e-07 (5.27e-07, 6.04e-07) |
|  | <35 | 3.31e-07 (2.70e-07, 3.92e-07) | <50 | 5.83e-07 (5.43e-07, 6.23e-07) |
|  | <30 | 6.45e-07 (5.67e-07, 7.23e-07) | <40 | 6.15e-07 (5.69e-07, 6.60e-07) |
|  | <25 | 9.65e-07 (8.50e-07, 1.08e-06) | <30 | 6.63e-07 (5.99e-07, 7.28e-07) |
|  | <20 | 9.52e-07 (7.59e-07, 1.15e-06) | <20 | 6.69e-07 (5.40e-07, 7.98e-07) |
|  | | | | |
| **moonRF** | | | | |
|  | **Long-term** | | **Short-term** | |
| PM_2.5_ (µg·m^-3^) | Full range | 3.53e-06 (3.29e-06, 3.76e-06) | Full range | 2.44e-07 (1.62e-07, 3.26e-07) |
|  | <14 | 3.54e-06 (3.31e-06, 3.78e-06) | <35 | 2.73e-07 (1.86e-07, 3.59e-07) |
|  | <13 | 3.66e-06 (3.42e-06, 3.90e-06) | <30 | 2.72e-07 (1.83e-07, 3.61e-07) |
|  | <12 | 4.01e-06 (3.75e-06, 4.27e-06) | <25 | 3.11e-07 (2.17e-07, 4.05e-07) |
|  | <11 | 4.46e-06 (4.16e-06, 4.77e-06) | <20 | 4.38e-07 (3.30e-07, 5.45e-07) |
|  | <10 | 4.64e-06 (4.26e-06, 5.03e-06) | <15 | 5.53e-07 (4.14e-07, 6.92e-07) |
|  | <9 | 4.35e-06 (3.77e-06, 4.93e-06) | <10 | 7.62e-07 (5.21e-07, 1.00e-06) |
|  | <8 | 5.46e-06 (4.53e-06, 6.39e-06) | <5 | 1.88e-06 (8.20e-07, 2.94e-06) |
|  | <7 | 5.15e-06 (3.43e-06, 6.88e-06) |  |  |
| O_3_ (ppb) | Full range | 2.43e-07 (1.02e-07, 3.84e-07) | Full range | 2.44e-07 (1.90e-07, 2.98e-07) |
|  | <42 | 5.65e-08 (-9.76e-08, 2.11e-07) | <100 | 2.47e-07 (1.92e-07, 3.01e-07) |
|  | <41 | 4.41e-08 (-1.20e-07, 2.08e-07) | <90 | 2.45e-07 (1.90e-07, 3.00e-07) |
|  | <40 | 1.74e-07 (-6.90e-09, 3.56e-07) | <80 | 2.38e-07 (1.83e-07, 2.94e-07) |
|  | <39 | 2.83e-07 (7.44e-08, 4.92e-07) | <70 | 2.49e-07 (1.91e-07, 3.07e-07) |
|  | <38 | 2.73e-07 (2.45e-08, 5.21e-07) | <60 | 2.32e-07 (1.67e-07, 2.98e-07) |
|  | <37 | 6.41e-07 (3.36e-07, 9.46e-07) | <50 | 3.60e-07 (2.68e-07, 4.52e-07) |
|  | <36 | 1.47e-06 (1.08e-06, 1.85e-06) | <45 | 5.78e-07 (4.53e-07, 7.03e-07) |
|  | <35 | 2.62e-06 (2.12e-06, 3.13e-06) | <40 | 5.82e-07 (3.93e-07, 7.71e-07) |
|  | <34 | 3.32e-06 (2.62e-06, 4.02e-06) | <35 | 4.65e-07 (1.44e-07, 7.87e-07) |
|  | <33 | 2.90e-06 (1.92e-06, 3.88e-06) | <30 | -1.67e-07 (-7.92e-07, 4.58e-07) |
|  | <32 | 2.23e-06 (8.32e-07, 3.63e-06) |  |  |
| NO_2_ (ppb) | Full range | 3.29e-07 (2.74e-07, 3.85e-07) | Full range | 5.56e-07 (5.17e-07, 5.94e-07) |
|  | <60 | 3.29e-07 (2.73e-07, 3.84e-07) | <100 | 5.56e-07 (5.18e-07, 5.94e-07) |
|  | <55 | 3.29e-07 (2.74e-07, 3.85e-07) | <90 | 5.56e-07 (5.18e-07, 5.95e-07) |
|  | <50 | 3.29e-07 (2.73e-07, 3.84e-07) | <80 | 5.55e-07 (5.17e-07, 5.94e-07) |
|  | <45 | 3.30e-07 (2.74e-07, 3.85e-07) | <70 | 5.55e-07 (5.16e-07, 5.93e-07) |
|  | <40 | 3.17e-07 (2.60e-07, 3.75e-07) | <60 | 5.60e-07 (5.21e-07, 5.99e-07) |
|  | <35 | 3.31e-07 (2.69e-07, 3.93e-07) | <50 | 5.79e-07 (5.39e-07, 6.19e-07) |
|  | <30 | 6.35e-07 (5.56e-07, 7.14e-07) | <40 | 6.10e-07 (5.64e-07, 6.55e-07) |
|  | <25 | 9.03e-07 (7.87e-07, 1.02e-06) | <30 | 6.45e-07 (5.80e-07, 7.10e-07) |
|  | <20 | 7.87e-07 (5.92e-07, 9.82e-07) | <20 | 6.65e-07 (5.36e-07, 7.94e-07) |

Numbers represent the probabilities of death (and 95% CIs) attribute to 1 µg·m^-3^ increase in PM_2.5_, 1 ppb increase in O_3_, or 1 ppb increase in NO_2_ at levels below increasingly stringent thresholds.

# **Section 9. Numeric results of sensitivity analysis of increasing bootstrap sample size to 620000 and the number of trees to 500 for moonRF**

| **moonRF** | | | | |
| --- | --- | --- | --- | --- |
|  | **Long-term** | | **Short-term** | |
| PM_2.5_ (µg·m^-3^) | Full range | 3.53e-06 (3.29e-06, 3.76e-06) | Full range | 2.43e-07 (1.61e-07, 3.25e-07) |
|  | <14 | 3.54e-06 (3.31e-06, 3.78e-06) | <35 | 2.72e-07 (1.86e-07, 3.59e-07) |
|  | <13 | 3.66e-06 (3.42e-06, 3.90e-06) | <30 | 2.72e-07 (1.83e-07, 3.60e-07) |
|  | <12 | 4.01e-06 (3.75e-06, 4.27e-06) | <25 | 3.10e-07 (2.16e-07, 4.04e-07) |
|  | <11 | 4.46e-06 (4.16e-06, 4.77e-06) | <20 | 4.38e-07 (3.30e-07, 5.45e-07) |
|  | <10 | 4.64e-06 (4.26e-06, 5.03e-06) | <15 | 5.54e-07 (4.15e-07, 6.93e-07) |
|  | <9 | 4.35e-06 (3.77e-06, 4.93e-06) | <10 | 7.68e-07 (5.26e-07, 1.01e-06) |
|  | <8 | 5.46e-06 (4.52e-06, 6.39e-06) | <5 | 1.93e-06 (8.66e-07, 2.99e-06) |
|  | <7 | 5.16e-06 (3.44e-06, 6.88e-06) |  |  |
| O_3_ (ppb) | Full range | 2.45e-07 (1.04e-07, 3.86e-07) | Full range | 2.45e-07 (1.91e-07, 2.99e-07) |
|  | <42 | 5.81e-08 (-9.60e-08, 2.12e-07) | <100 | 2.48e-07 (1.94e-07, 3.02e-07) |
|  | <41 | 4.56e-08 (-1.18e-07, 2.10e-07) | <90 | 2.47e-07 (1.92e-07, 3.01e-07) |
|  | <40 | 1.76e-07 (-4.68e-09, 3.58e-07) | <80 | 2.40e-07 (1.84e-07, 2.95e-07) |
|  | <39 | 2.86e-07 (7.73e-08, 4.95e-07) | <70 | 2.50e-07 (1.92e-07, 3.08e-07) |
|  | <38 | 2.76e-07 (2.79e-08, 5.25e-07) | <60 | 2.32e-07 (1.67e-07, 2.98e-07) |
|  | <37 | 6.45e-07 (3.40e-07, 9.50e-07) | <50 | 3.58e-07 (2.66e-07, 4.51e-07) |
|  | <36 | 1.47e-06 (1.08e-06, 1.86e-06) | <45 | 5.78e-07 (4.53e-07, 7.03e-07) |
|  | <35 | 2.62e-06 (2.11e-06, 3.13e-06) | <40 | 5.86e-07 (3.97e-07, 7.75e-07) |
|  | <34 | 3.31e-06 (2.61e-06, 4.01e-06) | <35 | 4.64e-07 (1.42e-07, 7.87e-07) |
|  | <33 | 2.90e-06 (1.92e-06, 3.88e-06) | <30 | -1.76e-07 (-8.02e-07, 4.49e-07) |
|  | <32 | 2.21e-06 (8.13e-07, 3.62e-06) |  |  |
| NO_2_ (ppb) | Full range | 3.31e-07 (2.75e-07, 3.86e-07) | Full range | 5.55e-07 (5.17e-07, 5.94e-07) |
|  | <60 | 3.30e-07 (2.75e-07, 3.86e-07) | <100 | 5.55e-07 (5.17e-07, 5.94e-07) |
|  | <55 | 3.31e-07 (2.75e-07, 3.86e-07) | <90 | 5.56e-07 (5.17e-07, 5.94e-07) |
|  | <50 | 3.30e-07 (2.74e-07, 3.86e-07) | <80 | 5.55e-07 (5.16e-07, 5.93e-07) |
|  | <45 | 3.31e-07 (2.75e-07, 3.87e-07) | <70 | 5.54e-07 (5.16e-07, 5.93e-07) |
|  | <40 | 3.19e-07 (2.62e-07, 3.76e-07) | <60 | 5.59e-07 (5.21e-07, 5.98e-07) |
|  | <35 | 3.33e-07 (2.71e-07, 3.95e-07) | <50 | 5.78e-07 (5.38e-07, 6.18e-07) |
|  | <30 | 6.38e-07 (5.59e-07, 7.17e-07) | <40 | 6.10e-07 (5.64e-07, 6.55e-07) |
|  | <25 | 9.09e-07 (7.94e-07, 1.02e-06) | <30 | 6.46e-07 (5.81e-07, 7.10e-07) |
|  | <20 | 8.05e-07 (6.10e-07, 1.00e-06) | <20 | 6.68e-07 (5.39e-07, 7.96e-07) |

Numbers represent the probabilities of death (and 95% CIs) attribute to 1 µg·m^-3^ increase in PM_2.5_, 1 ppb increase in O_3_, or 1 ppb increase in NO_2_ at levels below increasingly stringent thresholds.

# **Section 10. Positivity assessment**

The causal conclusion of this study replies on the key assumption of positivity, which requires that the density function of receiving any possible exposure is positive (1). However we cannot prove the lack of positivity with the observed data. Consequently, we categorized each exposure by the lower and upper percentiles (below 25th, 26th-74th, and above 75th) and compared the distributions of the estimated GPS across the exposure groups (2). The figure below shows the box plots between the estimated GPS and the categorized exposure levels:


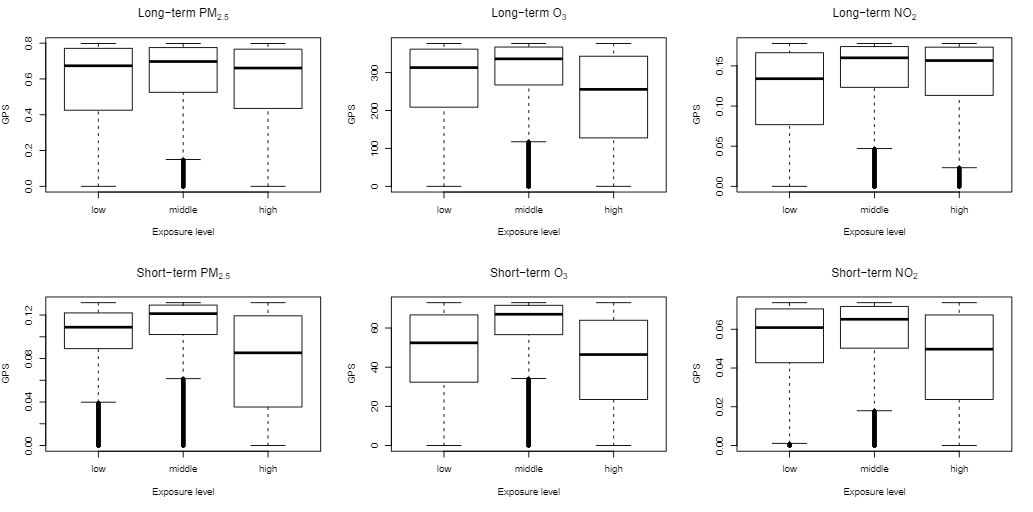


There are substantial overlaps across the box plots for each exposure, suggesting that the categorized exposure groups are relatively similar and the positivity assumption is likely to hold.

# **References**

1. Hirano K, Imbens GW. The Propensity Score with Continuous Treatments. In: Gelman A, Meng XL, editors. Applied Bayesian Modeling and Causal Inference from Incomplete‐Data. Hoboken, NJ: John Wiley & Sons, Ltd; 2004. p. 73-84.

2. McCaffrey DF, Griffin BA, Almirall D, Slaughter ME, Ramchand R, Burgette LF. A tutorial on propensity score estimation for multiple treatments using generalized boosted models. Stat Med. 2013;32(19):3388-414.
